# Genetic risk, incident colorectal cancer, and the benefits of adhering to a healthy lifestyle: A prospective study using data from UK Biobank and FinnGen

**DOI:** 10.3389/fonc.2022.894086

**Published:** 2022-10-06

**Authors:** E. Wu, Jun-Tao Ni, Xin Chen, Zhao-Hui Zhu, Hong-Quan Xu, Lin Tao, Tian Xie

**Affiliations:** ^1^ School of Pharmacy, Hangzhou Normal University, Hangzhou, China; ^2^ School of Public Health, Hangzhou Normal University, Zhejiang, China; ^3^ Scientific Research Department, Women’s Hospital School of Medicine Zhejiang University, Hangzhou, China; ^4^ Key Laboratory of Elemene Class Anti-Cancer Chinese Medicines, Engineering Laboratory of Development and Application of Traditional Chinese Medicines, Collaborative Innovation Center of Traditional Chinese Medicines of Zhejiang Province, Hangzhou Normal University, Hangzhou, China

**Keywords:** colorectal cancer, polygenic risk scores, lifestyle factors, epidemiology, prevention

## Abstract

**Background:**

Genetic factors increase the individual risk of colorectal cancer (CRC); however, the extent to which a healthy lifestyle can offset increased genetic risk is unknown. This study investigated whether a healthy lifestyle is associated with lower CRC risk, regardless of genetic risk.

**Methods:**

We recruited 390,365 participants without cancer at baseline (2006–2010) from the UK Biobank. The primary outcome was CRC incidence. A healthy lifestyle score constructed using 16 factors of six dimensions (smoking, drinking, body mass index, diet, exercise, and sleep) was categorized into three risk categories: favorable, intermediate, and unfavorable. To calculate the polygenic risk scores (PRSs) of UK Biobank participants, we extracted 454,678 single nucleotide polymorphisms (SNPs) from the UK Biobank and FinnGen Biobank after quality control. Cox proportional hazards regression was performed to evaluate the associations and was expressed as hazard ratios (HRs) with 95% confidence intervals (CIs).

**Results:**

During a median follow-up of 10.90 years, 4,090 new CRC cases were reported in the UK Biobank. The “best-fit” PRSs were constructed using 59 SNPs based on the UK Biobank cohort and FinnGen genome-wide association study summary data (R^2^ = 0.23%) and were divided into low (lowest quintile), intermediate (including second–fourth quintile), and high (highest quintile) genetic risk categories. The multivariate-adjusted Cox model revealed that participants with favorable lifestyles had HRs of 0.66 (95% CI = 0.60–0.72) for developing CRC *vs*. those with unfavorable lifestyles; low genetic risk was associated with a decreased risk of CRC (HR = 0.67, 95% CI =0.61–0.74) compared with those with high genetic risk. The HRs for low genetic risk participants with favorable lifestyles were 0.44 (95% CI =0.36–0.55) *vs*. participants with high genetic risk and unfavorable lifestyles. Among the participants with low, intermediate, or high genetic risk, the HRs of favorable *vs*. unfavorable lifestyles were 0.74, 0.64, and 0.72 (all *p*< 0.05).

**Conclusions:**

Low genetic risk and a favorable lifestyle were significantly associated with a decreased risk of CRC. A favorable lifestyle was associated with a lower CRC risk, regardless of genetic risk.

## Introduction

Colorectal cancer (CRC) is the third most common cancer and the fourth leading cause of cancer-related death worldwide (1). As a complex disease, CRC is determined by the interplay between genetic and lifestyle factors (2). Genome-wide association studies (GWASs) with large sample sizes have revealed that hundreds of independent loci are associated with CRC risk (3–10). Although each genetic variant contributes little to CRC, when aggregated into a polygenic risk score (PRS), these associated alleles can provide an overall measure of genetic susceptibility to certain diseases. Therefore, research on the relationship between CRC risk and PRS has attracted widespread attention.

The American Cancer Society (ACS) has emphasized the critical role of lifestyle management in CRC prevention (11). However, previous studies on CRC prevention have concentrated on limited factors, such as smoking, alcohol consumption, exercise, sedentary behavior, and intake of fruits and vegetables, while emerging factors, such as sleep and intake of whole and refined grains, have been excluded from the lifestyle scores. Owing to the increasing number of people with sleep disorders (12, 13), and sleep disorders have been shown to increase the risk of CRC (14–16). Incorporating emerging factors such as sleep into lifestyle scores to illustrate the benefits of a healthy lifestyle in CRC prevention should be addressed.

Therefore, this study aimed to evaluate the genetic risk of CRC among UK Biobank participants (created PRS based on GWAS summary data from the FinnGen biobank) and to determine whether adherence to a favorable lifestyle (including emerging factors such as sleep) is associated with a lower risk of CRC, regardless of genetic risk.

## Methods

### Study population

We obtained data from the UK Biobank and FinnGen cohort in this study. A detailed introduction to the UK Biobank has been provided elsewhere (https://www.ukbiobank.ac.uk/). Briefly, the UK Biobank collected and stored blood samples of approximately half a million volunteers from 2006 to 2010 and continually followed up their health and medical information. We extracted 502,414 participants from the UK Biobank. Exclusion criteria were (1) participants with missing values or without genotype data (n = 37,959); (2) nonwhite ethnic background (n = 27,385); and (3) with cancer at baseline, were diagnosed with CRC or died within the first 2 years of follow-up, or were lost to follow-up (n = 40,551). The present analyses were restricted to individuals from white ethnic backgrounds with available genetic and lifestyle information. After exclusion, 396,519 potentially eligible participants remained for further analyses (**Supplementary Figure S1**).

The CRC GWAS summary statistics in this study were obtained from the FinnGen Biobank and included 215,770 controls and 3,022 CRC cases (https://finngen.gitbook.io/documentation/). FinnGen data are openly available online. The need for ethical approval and consent to participate was waived, for they have been obtained from the original studies. The UK Biobank obtained ethical approval from the North West Multicenter Research Ethics Committee (reference no. 16/NW/0274). All the UK Biobank participants signed an informed consent form. This study was approved by the UK Biobank (no. 78563).

### Polygenic risk score

Polygenic risk score calculation requires separate base (GWAS summary statistics) and target data (individual-level genotype–phenotype data), as described previously (17, 18). For the base data, we used the FinnGen biobank GWAS summary data; for the target data, we used 396,519 individuals from the UK Biobank, which contains individual-level genotypes.

Both the base and target data require quality control. First, we filtered the base data of the FinnGen cohort. From a total of 16,380,465 single nucleotide polymorphisms (SNPs), we excluded 7,279,657 SNPs due to a minor allele frequency (MAF)< 1% (18). Second, regarding the target data of 396,519 UK Biobank participants, we selected version 2 of the non-imputed genotype data (continued to be correct and current) for the subsequent analysis (https://biobank.ctsu.ox.ac.uk/showcase/label.cgi?id=100319). A total of 784,256 SNPs located in autosomal chromosomes were used to calculate the PRS, and X chromosomes were used for sex checks (X chromosome homozygosity estimate of female individuals must be<0.2, and that of male individuals must be >0.8). The exclusion criteria were as follows (19): (1) missing ratio for SNPs and individuals >2%; (2) sex discrepancy; (3) MAF<1%; (4) Hardy–Weinberg equilibrium (HWE) *p*-value< 1×10^−6^ for controls and *p*-value< 1×10^−10^ for cases; (5) heterozygosity rate deviating more than triple the standard deviation (SD) from the mean; and (6) 10 or more third-degree relatives among participants. After quality control, there were 390,365 participants and 538,634 SNPs (located on autosomal chromosomes) for further analysis. Third, SNPs must be harmonized by assigning the same effect allele across the base and target datasets. SNPs with palindrome structures were removed. The remaining 390,365 individuals with 454,678 SNPs were used for PRS calculations (**Supplementary Figure S1**). PRS calculations were performed using PLINK 1.90 and PRSice-2 software (20). PRSice calculates PRS at numerous *p*-value thresholds to provide the best-fit PRS (21). The PRS was calculated according to the formula (20):


$$PRS=\sum_{i}^{m}\beta_{i}(\sum_{j=0}^{2}\omega_{ij}\times j)$$

where *m* is the number of SNPs under the corresponding *p*-value threshold, *β_i_* is *ln*(*OR_i_*)—natural logarithm transformed odds ratio of the *i*-th SNP obtained from the base data, and *ω*_ij_ indicates the probability of the genotype *j* (*j* = 0, 1, 2) for the *i*-th SNP.

### Healthy lifestyle scores

The lifestyle scores were established based on the ACS guidelines for CRC prevention, including exercise, alcohol consumption, body mass index (BMI), and diet (intake of fruits, vegetables, fish, whole grains, processed meat, red meat, and refined grains) (11). In addition, we included sleep (sleep duration, chronotype, insomnia, snoring, and daytime dozing) and smoking in the lifestyle scores because of the growing interest in the association of sleep disorders and smoking with higher CRC risk (14, 15, 22, 23). Information on lifestyle factors was obtained at baseline by using a validated touchscreen questionnaire. Participants accumulated 1 point once in accordance with each of following six healthy lifestyle patterns based on ACS guidelines or national recommendations (**Supplementary Table S1**): 1) never smoked (24); 2) moderate alcohol consumption [women ≤ 14 g/day or men ≤ 28 g/day (25)]; 3) regular exercise (moderate physical activities ≥ 150 min/week, or ≥ 5 days/week, or vigorous exercise ≥ 75 min/week, or ≥ once a week, or an equivalent combination (26, 27); 4) healthy diet (individuals incorporating at least three of the following dietary behaviors: fruits ≥ 3 servings/day, vegetables ≥ 3 servings/day, fish ≥ twice a week, processed meats ≤ once a week, red meats ≤ twice a week, whole grains ≥ 3 servings/day, refined grains ≤ 1 serving/day) (28); 5) sleeping well (individuals having at least three of the following healthy sleep behaviors: sleep duration, 7–8 h/day, chronotype indicated as morning person, never or sometimes insomnia, no snoring, never or sometimes daytime dozing) (29); and 6) normal weight, 18.5 kg/m^2^ ≤ body mass index (BMI)< 25.0 kg/m^2^ is considered healthy weight (30). The total healthy lifestyle score ranges from 0 to 6, and the higher the score, the higher the tendency for a healthy lifestyle. Healthy lifestyle scores were categorized into three groups: unfavorable (scored 0–2 points), intermediate (scored 3–4 points), and favorable (scored 5-6 points).

### Covariates

Covariate information was obtained at baseline, including the age when attending the assessment center; sex (female, male); ethnic background (British, Irish, any other white background); socioeconomic status as measured using the Townsend deprivation index (TDI), which combining information on non-homeownership, household overcrowding, non-car ownership, and unemployment at recruitment (31); and education (higher: college or university; middle: A levels/AS levels or equivalent, O levels/GCSEs or equivalent; lower: CSEs or equivalent, NVQ, HND, HNC, or equivalent; vocational: professional qualifications, e.g., nursing, teaching; other).

### Ascertainment of CRC outcome

The primary outcome was the first incident CRC diagnosis or CRC first documented on the death certificates. Death information from the UK Biobank was obtained from the “death registry,” which is linked to the National Health Service (NHS) Central Register (Scotland participants) and NHS Digital Center (England/Wales participants) (32). Participants who died of CRC were recorded by the 10th revision of the International Classification of Diseases (ICD-10) (coding: C18, C19, and C20). The date of the “death registry” was updated to 31 October 2021 (for Scotland participants) or 30 September 2021 (for England/Wales participants).

The details of the first incident CRC diagnosis were obtained from the “cancer registry,” which is linked to the Information Services Division of the NHS Scotland (Scotland participants) and the Medical Research Information Service of the NHS (England/Wales participants) (33). CRC incidence was diagnosed using ICD-10 (C18, C19, and C20), the ICD-9 (153, 1540, and 1541), and self-reported cancer (1020, 1022, and 1023). The follow-up person-years of each participant were calculated from the baseline survey time to either the date of CRC outcome, death for any reason, 31 October 2015 (for Scotland participants), or 29 February 2020 (for England/Wales participants), whichever occurred first.

### Statistical analysis

The baseline characteristics of each participant are presented by CRC status as frequencies (percentages) for categorical variables and median [(interquartile range) (IQR)] for non-normally distributed continuous variables. Pearson’s χ^2^ test was used to analyze unordered categorical variables, and the Wilcoxon rank test was used to analyze grade variables or non-normally distributed continuous variables. The reverse Kaplan–Meier method was used to calculate the median follow-up time.

Cox proportional hazards regression was used to evaluate the associations of genetic, lifestyle, and combined genetic–lifestyle factors with the risk of CRC; it was adjusted for potential confounding factors, and the results were expressed as adjusted hazard ratios (HRs) with 95% confidence intervals (CIs). Model 1 was adjusted for age (years), sex (male and female), education (higher, middle, lower, vocational, and other), Townsend deprivation index, and the first 10 genetic principal components (GPCs) at recruitment; Model 2 was additionally adjusted for healthy lifestyle scores (0-6), and Model 2b was additionally adjusted for the PRS based on Model 1. The proportionality of hazards assumption was examined using the Schoenfeld residuals; no evidence of non-proportionality was observed. Multiplicative interactions were calculated using Cox regression adjusted for Model 1. A restricted cubic spline (RCS) using three knots was used to examine dose–response associations. Additionally, absolute risks (ARs) were calculated using the CRC incidence in each risk group (34, 35).

Sensitivity analysis was conducted to examine the robustness of the findings. First, stratified analyses were conducted according to age at baseline, sex, socioeconomic status, and education. Second, weighted lifestyle scores were created and classified into three groups: unfavorable, intermediate, and favorable. Statistical analyses were performed using SAS version 9.1 (SAS Institute, Cary, NC, USA), and graphs were plotted using R [version 4.1.3; R Core Team (2022); Vienna, Austria]. All *p*-values were two-sided, and a *p*-value< 0.05 was considered statistically significant.

## Results

The baseline characteristics of the 390,365 participants who were followed up for a median of 10.90 years (follow-up 4,135,798 total person-years) are presented by CRC status in **Table 1**. There were 4,090 cases of CRC. Participants without CRC were younger, more likely to be female, had a healthy diet, had normal weight, and never smoked (*p*< 0.05) (**Supplementary Table S2**). Among 454,678 SNPs for PRS calculation, we found that a *p*-value< 5×10^−5^ threshold, containing 59 SNPs, generated a “best-fit” PRS (R^2^ = 0.23%) (**Supplementary Figure S2**). The “best-fit” PRS and healthy lifestyle score (from 0 to 6 points) were all in accordance with a normal distribution (**Supplementary Figure S3**). The dose–response relationship suggested a positive association between PRS and CRC risk (**Supplementary Figure S4**). The PRS was then categorized into three genetic risk groups: low (lowest quintile), intermediate (including second–fourth quintile), and high (highest quintile). Notably, genetic risk categories were not associated with any lifestyle factors except for smoking status and exercise (**Supplementary Table S3**).

**Table 1:** Baseline characteristics of the study participants by CRC status.

Characteristic	Incident CRC		*p*-value
	No (n = 386275)	Yes (n = 4090)	
Age, median (IQR)^a^	57 (50,63)	62 (57,66)	<0.001
Sex^b^			<0.001
Female	206,991 (53.6)	1,739 (42.5)	
Male	179,284 (46.4)	2,351 (57.5)	
TDI^a^			0.392
1 (least deprived)	77,246 (20.0)	851 (20.8)	
2–4 (intermediate deprived)	231,783 (60.0)	2,441 (59.7)	
5 (most deprived)	77,246 (20.0)	798 (19.5)	
Education^b^			<0.001
Higher	127,986 (33.1)	1,247 (30.5)	
Middle	129,664 (33.6)	1,326 (32.4)	
Lower	46,887 (12.1)	497 (12.2)	
Vocational	19,675 (5.1)	224 (5.5)	
Others	62,063 (16.1)	796 (19.5)	
Healthy lifestyle factors^b,c^			
Healthy diet	194,537 (50.4)	1,985 (48.5)	0.020
Normal weight	127,880 (33.1)	1,079 (26.4)	<0.001
Never smoked	211,193 (54.7)	1,879 (45.9)	<0.001
Regular exercise	284,657 (73.8)	2,919 (71.4)	0.001
Sleep well	234,927 (60.8)	2,350 (57.5)	<0.001
Moderate drinking	279,395 (72.3)	2,797 (68.4)	<0.001
No. of healthy lifestyle scores^a^			<0.001
0	4,225 (1.1)	58 (1.4)	
1	25,326 (6.6)	381 (9.3)	
2	64,567 (16.7)	860 (21.0)	
3	100,695 (26.1)	1,131 (27.7)	
4	103,037 (26.7)	946 (23.1)	
5	66,654 (17.3)	553 (13.5)	
6	21,771 (5.6)	161 (3.9)	
Genetic risk category^a^			<0.001
Low	77,382 (20.0)	687 (16.8)	
Intermediate	231,837 (60.0)	2,386 (58.3)	
High	77,056 (19.9)	1,017 (24.9)	

a The Wilcoxon rank test.

b Pearson’s χ^2^ test.

c Binary variable, showing one of the options.

TDI, Townsend deprivation index; due to the rounding off, the column percentages for each item may not be 100.

The risk of CRC decreased monotonically across the healthy lifestyle groups (**Table 2**). Participants with a favorable lifestyle had an HR of 0.66 (95% CI = 0.60–0.72), and those with an intermediate lifestyle had an HR of 0.77 (95% CI =0.72–0.83) for developing CRC *vs*. those with an unfavorable lifestyle in Model 1. The results remained significant after additional adjustment for PRS in Model 2. The HRs for favorable and intermediate lifestyles were 0.66 (95% CI = 0.60–0.72) and 0.78 (95% CI = 0.72–0.83), respectively. A similar pattern of effects was observed when healthy lifestyle scores were used as continuous variables (**Supplementary Table S4**).

**Table 2:** Risk of CRC according to lifestyle category.

Model	Lifestyle category			*p*-value for trend
	Unfavorable (n = 95,417)	Intermediate (n = 205,809)	Favorable (n = 89,139)	
No. of CRC/ person-years	1,299/1,003,622	2,077/2,182,758	714/949,418	
Absolute risk	1.4%	1.0%	0.8%	
HR (95% CI)^a^	1.00 (ref)	0.77 (0.72–0.83)	0.66 (0.60–0.72)	<0.001
HR (95% CI)^b^	1.00 (ref)	0.78 (0.72–0.83)	0.66 (0.60–0.72)	<0.001

CRC, colorectal cancer; HR, hazard ratio; CI, confidence interval.

a Model 1, Cox proportional hazards regression adjusted for age, sex, education, Townsend deprivation index, and first 10 genetic principal components.

b Model 2, Cox proportional hazards regression adjusted for Model 1 and polygenic risk scores.

p-value for trend was modeling the lifestyle categories as a continuous variable.

A decreased monotonically gradient risk of CRC was also observed across low genetic risk groups (**Table 3**). Participants with low genetic risk had HRs of 0.67 (95% CI = 0.61–0.74), and intermediate genetic risk participants had HRs of 0.78 (95% CI =0.73–0.84) for developing CRC *vs*. high genetic risk participants. The AR for CRC was 1.3% in the high-risk genetic group and 0.9% in the low-risk genetic group. In addition, the higher PRS quintiles were also associated with a monotonically increased risk of CRC compared with the lowest quintile (**Supplementary Table S5**).

**Table 3:** Risk of CRC according to genetic risk.

Model	Genetic category			*p*-valuefor trend
	High (n = 78,073)	Intermediate (n = 234,223)	Low (n = 78,069)	
No. of CRC/ person-years	1,017/824,989	2,386/2,482,760	687/828,049	
Absolute risk	1.3%	1.0%	0.9%	
HR (95% CI)	1.00 (ref)	0.78 (0.73–0.84)	0.67 (0.61–0.74)	<0.001

CRC, colorectal cancer; HR, hazard ratio; CI, confidence interval.

Cox proportional hazards regression adjusted for age, sex, education, Townsend deprivation index, first 10 genetic principal components, and healthy lifestyle scores. p-value for trend was modeling the lifestyle categories as a continuous variable.

To explore the impact of lifestyle on CRC risk according to different genetic risk categories, we stratified lifestyles by genetic risk category using unfavorable lifestyles as the reference group and found that favorable lifestyles were associated with a lower risk of CRC in any genetic risk group (**Table 4**). In the low genetic risk category, participants with a favorable lifestyle had an HR of 0.74 (95% CI = 0.51–0.81) for developing CRC *vs*. participants with an unfavorable lifestyle. The HRs of favorable lifestyles *vs*. unfavorable lifestyles among participants in intermediate and high genetic risk category were 0.64 (95% CI = 0.56–0.72) and 0.72 (95% CI = 0.60–0.87), respectively.

**Table 4:** Risk of CRC according to lifestyle category within each PRS category.

	High genetic risk			Intermediate genetic risk			Low genetic risk		
Lifestyle category	Unfavorable (n = 19,043)	Intermediate (n = 41,249)	Favorable (n = 17,781)	Unfavorable (n = 57,508)	Intermediate (n = 123,359)	Favorable (n = 53,356)	Unfavorable (n = 18,866)	Intermediate (n = 41,201)	Favorable (n = 18,802)
No. of CRC cases/ person-years	305/199,687	526/436,389	186/188,913	785/605,264	1,188/1,309,033	413/568,462	209/198,670	363/437,336	115/192,042
Absolute risk	1.6%	1.3%	1.0%	1.4%	1.0%	0.8%	1.1%	0.9%	0.6%
HR (95% CI)^a^	1.00 (ref)	0.83 (0.72–0.96)	0.72 (0.60–0.87)	1.00 (ref)	0.74 (0.67–0.81)	0.64 (0.56–0.72)	1.00 (ref)	0.83 (0.70–0.98)	0.74 (0.51–0.81)
*p*-value		0.012	0.001		<0.001	<0.001		0.030	<0.001
*p*-value for trend	0.002			<0.001			0.001		

CRC, colorectal cancer; HR, hazard ratio; CI, confidence interval.

a Cox proportional hazards regression adjusted for age, sex, education, Townsend deprivation index, and first 10 genetic principal components.

**Figure 1:**
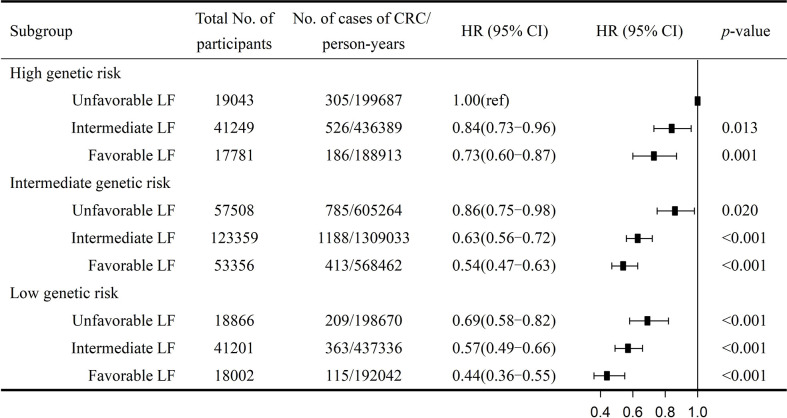
Risk of CRC according to genetic and lifestyle risk. LF, lifestyle; HR, hazard ratio; CI, confidence interval. Cox proportional hazards regression adjusted for age, sex, education, Townsend deprivation index, and the first 10 principal components of ancestry. *p* for multiplicative interaction< 0.001.

We further combined the genetic and lifestyle categories to explore the joint effects of genetic–lifestyle on CRC risk. As shown in **Figure 1**, participants with low genetic risk and favorable lifestyles had an HR of 0.44 (95% CI =0.36–0.55) for developing CRC *vs*. participants with high genetic risk and unfavorable lifestyles. The HRs for low genetic risk participants with unfavorable lifestyles were 0.69 (95% CI =0.58–0.82) *vs*. participants with high genetic risk and unfavorable lifestyles.

The sensitivity analysis indicated that the results were robust. First, low genetic risk combined with a healthy lifestyle has been observed to be associated with lower CRC risk when stratified by age, sex, socioeconomic status, and education (**Supplementary Tables S6**-**S9**). Second, the results did not change when using the weighted lifestyle scores (**Supplementary Figures S5**, **S6**).

## Discussion

This study presented the quantitative effects of genetic risk, lifestyle, and their joint effects on CRC risk using data from the UK Biobank and FinnGen. The results indicated that the high genetic risk was significantly correlated with an increased CRC risk. If individuals were at high genetic risk for CRC, the susceptibility of CRC may be enhanced but may also be modified through a healthy lifestyle, namely, as a result of their combined effects. Notably, adherence to a favorable lifestyle was correlated with a lower CRC risk, regardless of the genetic risk category.

Numerous studies have reported that decreased CRC risk is related to a healthy lifestyle, which is consistent with the results of this study (36–38). For instance, a Danish cohort study involving 55,487 participants constructed a lifestyle score for factors such as smoking, exercise, drinking, waist circumference, and diet and found that a healthy lifestyle was associated with reduced CRC risk (RR = 0.89) (39). Compared with past studies, our research scale was an order of magnitude larger, and genetic risk was considered. We selected 59 SNPs from 454,678 SNPs to construct the “best-fit” PRS, which can effectively evaluate the genetic susceptibility of CRC and help save costs. Although individuals may think that they are powerless against their CRC genetic susceptibility, our research shows that favorable lifestyles can still significantly lower the risk of CRC, regardless of genetic background. Within any genetic risk background, adherence to a favorable lifestyle may modify CRC risk and, to a certain extent, help to prevent CRC.

In recent years, studies on the genetic and lifestyle risks of CRC have begun. A recent study of 346,297 UK Biobank participants constructed PRS and a healthy lifestyle score including eight lifestyle factors (BMI, waist-to-hip ratio, exercise, sedentary time, intake of processed meat, red meat, vegetables, and fruits) and found that the PRS and healthy lifestyle had a significant additive interaction (*p*< 0.05) (40). However, the base data (containing 95 SNPs) used in that study were derived from two published articles, partially involving East Asians and African Americans (35, 41), and the lifestyle factors included in the analysis were not comprehensive enough. In our study, we used the entire FinnGen cohort (containing 16,380,465 SNPs) as the base data; the Finnish and UK populations are genetically closer compared to the UK population and East Asians and African Americans, and the SNPs selected to construct the PRS are also different. In addition, we included sleep duration; sleep chronotype; insomnia; snoring; daytime dozing; and intake of fish, whole grains, and refined grains in the construction of a healthy lifestyle score. Our study provides evidence of a healthy lifestyle for lower CRC risk, regardless of genetic background. Our findings are consistent with a case–control analysis using 7,558 German participants, which investigated the absolute CRC risk according to genetics, diet, BMI, smoking, alcohol consumption, exercise, and colonoscopy history, and found that the OR for high genetic risk was about 2.20 *vs*. that for the low genetic risk, and the OR for a favorable lifestyle was about 0.50 *vs*. that for an unfavorable lifestyle (3). Our research included six dimensions of 16 lifestyle factors and provided quantitative results to complement the joint effects of genetic risk and lifestyle factors on CRC. These results revealed a strong association of genetic and lifestyle factors with the risk of CRC. Our study supports the long-held view that genetic variants identifiable at birth change the risk of CRC (42–44), which is beneficial for the primary prevention of CRC.

Numerous biological mechanisms have been proposed to explain the association of genetics and lifestyle with the risk of CRC. Insufficient exercise affects the body’s immune system and depresses immune function in CRC, while nicotine may partially promote colorectal tumor growth and angiogenesis (45). Excess drinking causes acetaldehyde to accumulate in the colorectum, which may cause DNA damage (46). Inadequate sleep may affect the release of prolactin, growth hormone, and melatonin and may also depress the function of proinflammatory cytokine genes (47). These unhealthy lifestyles may lead to obesity, systemic inflammation, insulin resistance, and type 2 diabetes and may impact CRC risk to a certain extent. The genetic risk of CRC may be related to mutated oncogenes, such as APC, TP53, KRAS, PI3KCA, BRAF, and NRAS (48, 49). Our study found that there is a high risk of CRC in high genetic risk categories with unfavorable lifestyles, which may better alert the high-risk CRC population to adopt a healthy lifestyle.

This is the largest prospective cohort study to date that has investigated the association of genetic factors and lifestyles, including six dimensions, with CRC risk. It focuses on the risk of integrated lifestyles and genetic susceptibility to CRC. Compared to modeling a single factor alone, it can better capture the complex nature and multiple dimensions of lifestyle behaviors. However, this study has some limitations. First, lifestyle scores use a dichotomy, and the selection of cutoff points is mainly based on public health recommendations, which are general rather than specific for certain risks. Second, several participants were excluded because of missing values, which may have significantly reduced the data. Third, the lifestyle factors that we constructed did not include all possible lifestyle factors such as aspirin medication history, family history of CRC, and other possible influencing factors. Incorporating them into the model may help improve the accuracy of CRC risk assessment. Finally, this study mainly focused on participants from a white ethnic background. Future studies should evaluate the generalizability of these research results to other populations.

## Conclusions

In conclusion, our findings validate that low genetic risk and a favorable lifestyle are significantly associated with a decreased CRC risk. Additionally, we found that a favorable lifestyle was associated with a lower CRC risk in any genetic background, indicating the potential of modifiable lifestyle factors in CRC prevention.

## Data availability statement

Publicly available datasets were analyzed in this study. The data can be found here: The FinnGen cohort Genome-wide association study (GWAS) summary data are openly available from the website https://finngen.gitbook.io/documentation/(R5). The UK Biobank datasets are openly available by submitting a data request proposal from https://www.ukbiobank.ac.uk/. We are able to access the UK Biobank database through the Access Management System (AMS). The application number is 78563.

## Author contributions

EW performed the statistical analysis and wrote the manuscript. J-TN designed the study. XC, Z-HZ, and H-QX were responsible for technical support. TX and LT revised the manuscript. All authors contributed to the article and approved the submitted version.

## Funding

This research was funded by the General Projects of National Natural Science Foundation of China (81973635) (to TX) and the Start-up Grant of HZNU (4125C5021720421) (to LT). The funding sources had no role in the study design; in the collection, analysis, or interpretation of data; in the writing of the report; or in the decision to submit the article for publication.

## Acknowledgments

We thank the participants and staff of the UK Biobank for their dedication and contribution to the research.

## Conflict of interest

The authors declare that the research was conducted in the absence of any commercial or financial relationships that could be construed as a potential conflict of interest.

The reviewer XC declared a shared parent affiliation with the author JN to the handling editor at the time of review.

## Publisher’s note

All claims expressed in this article are solely those of the authors and do not necessarily represent those of their affiliated organizations, or those of the publisher, the editors and the reviewers. Any product that may be evaluated in this article, or claim that may be made by its manufacturer, is not guaranteed or endorsed by the publisher.

## Abbreviations

CRC, colorectal cancer; SNP, single nucleotide polymorphism; PRS, polygenic risk scores; HR, hazard ratio; CI, confidence interval; GWAS, genome-wide association study; GPC, genetic principal component; MAF, minor allele frequency; HWE, Hardy–Weinberg equilibrium; SD, standard deviation; BMI, body mass index; TDI, Townsend deprivation index.
