# Supplementary material for: Genetic risk, incident colorectal cancer, and the benefits of adhering to a healthy lifestyle: A prospective study using data from UK Biobank and FinnGen
Source: Front Oncol. 2022 Oct 6;12:894086. doi: 10.3389/fonc.2022.894086 (PMC9582975; doi:10.3389/fonc.2022.894086)
Supplement: Supplementary file 1 [file DataSheet_1.pdf]

Supplementary Material

Supplementary Table 1 Healthy lifestyle factors definitions
Supplementary Table 2 CRC risk according to each lifestyle factors
Supplementary Table 3 Associations between the polygenic risk score and individual lifestyle factors
Supplementary Table 4 CRC risk according to healthy lifestyle scores
Supplementary Table 5 CRC risk according to genetic risk quintile
Supplementary Table 6 CRC risk according to genetic and lifestyle risk stratified by age
Supplementary Table 7 CRC risk according to genetic and lifestyle risk stratified by sex
Supplementary Table 8 CRC risk according to genetic and lifestyle risk stratified by socioeconomic status
Supplementary Table 9 CRC risk according to genetic and lifestyle risk stratified by education

Supplementary Figure 1 Cohort exclusions of the study participants
Supplementary Figure 2 PRSice-2 showing results at broad *p*-value thresholds for PRS predicting CRC
Supplementary Figure 3 Distribution of the polygenic risk scores and healthy lifestyle scores
Supplementary Figure 4 Multivariable-adjusted dose-response associations between PRS and CRC risk
Supplementary Figure 5 Risk of CRC according to weighted lifestyle category
Supplementary Figure 6 Risk of CRC according to genetic and weighted lifestyle categories

Supplementary Table 1 Healthy lifestyle factors definitions

Lifestyle	Coding	Healthy lifestyle definition and calculation methods.
Never Smoked	20116	**Never smoked** defined as a healthy level will score 1 point.
Moderate drinking		**Females who consumed alcohol ≤ 14 g/d or males ≤ 28 g/day** defined as moderate drinking will score 1 point. Alcohol content = alcohol capacity (ml) × alcohol degree × 0.8. We described 1 serving as 14g to facilitate calculations.
	1558,20117	Never equals to 0 servings.
	1568, 4407,	125ml red wine equals to 0.85 servings.
	4418,1578,	125ml white wine equals to 0.85 servings.
	1588, 4429	568 ml beer equals to 1.28 servings.
	1598, 4440	25ml spirits equals to 0.57 servings.
	1608, 4451	50ml fortified wine equals to 0.56 servings.
	4462, 5364	125ml other alcoholic equals to 0.36 servings.
Sleep well		Five healthy sleep behaviors below, Participants will score 1 point if they have ≥ 3 healthy sleep behavior:
	1160	**Sleep duration: 7−8 h/d**
	1180	**Chronotype: morning person.**
	1200	**Insomnia: never or sometimes**
	1210	**Snoring：no**
	1220	**Daytime dozing: never or sometimes**
Healthy diet		The following seven dietary behaviors meet the recommended intake according to the guidelines of US diet; Participants will score 1 point if they have ≥ 3 healthy diet behaviors as below.
	1309, 1319	**Fruits ≥ 3 servings/day.**
	1289, 1299	**Vegetables ≥ 3 servings/day.**
	1329, 1339	**Fish ≥ twice a week.**
	1448,1438	**Whole grains ≥ 3 servings/day**
	1349	**Processed meat ≤ once a week.**
	1369,1379,1389	**Red meat ≤ twice a week**
	1458, 1468	**Refined grains ≤ 1 servings/day**
Regular exercise		If the participant's single item or equivalent combination met any of the following exercise items will define as **regular exercise** and scored 1 point.
	884	Moderate physical activity ≥ 5 days a week.
	884*894	Moderate physical activity ≥ 150 minutes a week
	904	Vigorous activity ≥ once a week
	904*914	Vigorous activity ≥ 75 minutes a week
	884*894+904*914*2	Physical activity ≥ 150 minutes a week
Normal weight	21001,23104	18.5 kg/m^2 ≤ Body mass index < 25.0 kg/m^2 defined as **normal weight** will score 1 point.

Supplementary Table 2 CRC risk according to each lifestyle factors

Characteristic	Incident CRC		β	HR (95% CI)	p
	No (n = 386275)	Yes (n = 4090)			
Diet					
Unhealthy	191738(49.6)	2105(51.5)		1.00(ref)	
Healthy	194537(50.4)	1985(48.5)	-0.085	0.92(0.86-0.98)	0.008
Weight					
Abnormal	258395(66.9)	3011(73.6)		1.00(ref)	
Normal	127880(33.1)	1079(26.4)	-0.189	0.83(0.77-0.89)	<0.001
Smoking status					
Current or former	175082(45.3)	2211(54.1)		1.00(ref)	
Never smoke	211193(54.7)	1879(45.9)	-0.219	0.80(0.75-0.86)	<0.001
Exercise					
Insufficient	101618(26.3)	1171(28.6)		1.00(ref)	
Regular	284657(73.8)	2919(71.4)	-0.131	0.88(0.82-0.94)	<0.001
Sleep					
Disorder	151348(39.2)	1740(42.5)		1.00(ref)	
Well	234927(60.8)	2350(57.5)	-0.113	0.89(0.84-0.95)	<0.001
Drinking					
Over-drinking	106880(27.7)	1293(31.6)		1.00(ref)	
Moderate	279395(72.3)	2797(68.4)	-0.195	0.82(0.77-0.88)	<0.001

Abbreviation: CRC, colorectal cancer; HR, hazard ratio; CI, confidence interval.

Cox proportional hazards regression adjusted for age, sex, education, Townsend deprivation index and first 10 genetic principal components.

Supplementary Table 3 Associations between the polygenic risk score and individual lifestyle factors

Characteristic	Genetic risk			*p* value
	High (n = 78073)	Intermediate (n = 234223)	low (n = 78069)	
Diet				0.132
Unhealthy	38703(49.6)	116592(49.8)	38548(49.4)	
Healthy	39370(50.4)	117631(50.2)	39521(50.6)	
Weight				0.844
Abnormal	52349(67.1)	156805(66.9)	52252(66.9)	
Normal	25724(32.9)	77418(33.1)	25817(33.1)	
Smoking status				0.008
Current or former	35505(45.5)	106715(45.6)	35073(44.9)	
Never smoking	42568(54.5)	127508(54.4)	42996(55.1)	
Exercise				0.005
Insufficient	20704(26.5)	61882(26.4)	20203(25.9)	
Regular	57369(73.5)	172341(73.6)	57866(74.1)	
Sleep				0.923
Disorder	30574(39.2)	91866(39.2)	30648(39.3)	
Well	47499(60.8)	142357(60.8)	47421(60.7)	
Drinking				0.478
Over-drinking	21750(27.9)	64749(27.6)	21674(27.8)	
Moderate	56323(72.1)	169474(72.4)	56395(72.2)	

Pearson's $\chi 2$ test

Supplementary Table 4 CRC risk according to healthy lifestyle scores

Unhealthy lifestyle score	HR (95% CI)	*p*-value	*p*-value for trend
0 healthy lifestyle factor	1.00 (ref)		
1 healthy lifestyle factor	1.09(0.83-1.44)	0.539	
2 healthy lifestyle factors	0.98(0.75-1.27)	0.852	
3 healthy lifestyle factors	0.85(0.65-1.10)	0.211	<0.001
4 healthy lifestyle factors	0.71(0.55-0.93)	0.012	
5 healthy lifestyle factors	0.67(0.51-0.88)	0.004	
6 healthy lifestyle factors	0.62(0.46-0.84)	0.002	

Abbreviation: CRC, colorectal cancer; HR, hazard ratio; CI, confidence interval.

Cox proportional hazards regression adjusted for age, sex, education, Townsend deprivation index and first 10 genetic principal components.

Supplementary Table 5 CRC risk according to genetic risk quintile

Genetic risk quintiles	HR (95% CI)	*p*-value	*p*-value for trend
Quintile 1 (lowest)	1.00 (ref)		
Quintile 2	1.09(0.99-1.21)	0.094	
Quintile 3	1.14(1.03-1.26)	0.013	<0.001
Quintile 4	1.27(1.15-1.41)	<0.001	
Quintile 5 (highest)	1.49(1.35-1.64)	<0.001	

Abbreviation: CRC, colorectal cancer; HR, hazard ratio; CI, confidence interval.

Cox proportional hazards regression adjusted for age, sex, education, Townsend deprivation index and first 10 genetic principal components.

Supplementary Table 6 CRC risk according to genetic and lifestyle risk stratified by age

	40-54 years (n = 153297)	55-64 years (n = 166421)	≥65 years (n = 70647)
Subgroup	HR (95% CI)	HR (95% CI)	HR (95% CI)
High genetic risk			
Unfavorable LF	1.00 (ref)	1.00 (ref)	1.00 (ref)
Intermediate LF	0.90(0.65-1.25)	0.72(0.59-0.88)	1.02(0.79-1.32)
Favorable LF	0.85(0.57-1.26)	0.68(0.52-0.88)	0.76(0.54-1.07)
Intermediate genetic risk			
Unfavorable LF	0.79(0.57-1.08)	0.82(0.68-0.98)	0.97(0.76-1.24)
Intermediate LF	0.63(0.47-0.85)	0.59(0.50-0.70)	0.71(0.56-0.90)
Favorable LF	0.67(0.48-0.93)	0.45(0.37-0.56)	0.62(0.47-0.82)
Low genetic risk			
Unfavorable LF	0.59(0.38-0.91)	0.66(0.52-0.85)	0.80(0.59-1.10)
Intermediate LF	0.62(0.44-0.88)	0.53(0.43-0.66)	0.62(0.47-0.83)
Favorable LF	0.27(0.15-0.49)	0.43(0.32-0.58)	0.60(0.42-0.87)
p value for trend	<0.001	<0.001	<0.001

Abbreviation: CRC, colorectal cancer; HR, hazard ratio; CI, confidence interval; LF, lifestyle.

Cox proportional hazards regression adjusted for age, sex, education, Townsend deprivation index and first 10 genetic principal components.

Supplementary Table 7 CRC risk according to genetic and lifestyle risk stratified by sex

	Female (n = 208730)		Male (n = 181635)	
Subgroup	HR (95% CI)	*p* value	HR (95% CI)	*p* value
High genetic risk				
Unfavorable LF	1.00 (ref)		1.00 (ref)	
Intermediate LF	0.79(0.63-0.99)	0.045	0.86(0.71-1.02)	0.089
Favorable LF	0.77(0.59-1.01)	0.056	0.61(0.46-0.81)	0.001
Intermediate genetic risk				
Unfavorable LF	0.71(0.56-0.89)	0.004	0.93(0.79-1.09)	0.379
Intermediate LF	0.57(0.46-0.71)	<0.001	0.67(0.57-0.78)	<0.001
Favorable LF	0.55(0.44-0.69)	<0.001	0.50(0.40-0.61)	<0.001
Low genetic risk				
Unfavorable LF	0.57(0.42-0.79)	0.001	0.74(0.60-0.92)	0.006
Intermediate LF	0.55(0.43-0.70)	<0.001	0.58(0.47-0.70)	<0.001
Favorable LF	0.44(0.33-0.60)	<0.001	0.42(0.31-0.58)	<0.001
p value for trend	<0.001		<0.001	

Abbreviation: CRC, colorectal cancer; HR, hazard ratio; CI, confidence interval; LF, lifestyle.

Cox proportional hazards regression adjusted for age, sex, education, Townsend deprivation index and first 10 genetic principal components.

Supplementary Table 8 CRC risk according to genetic and lifestyle risk stratified by socioeconomic status

	TDI quintile 1 (n = 78097)	TDI quintile 2-4 (n = 234224)	TDI quintile 5 (n = 78044)
Subgroup	HR (95% CI)	HR (95% CI)	HR (95% CI)
High genetic risk			
Unfavorable LF	1.00 (ref)	1.00 (ref)	1.00 (ref)
Intermediate LF	0.76(0.55-1.03)	0.81(0.67-0.98)	1.00(0.74-1.34)
Favorable LF	0.59(0.40-0.88)	0.77(0.61-0.98)	0.74(0.47-1.15)
Intermediate genetic risk			
Unfavorable LF	0.84(0.63-1.13)	0.88(0.74-1.05)	0.81(0.61-1.07)
Intermediate LF	0.63(0.48-0.83)	0.63(0.53-0.74)	0.63(0.49-0.83)
Favorable LF	0.49(0.36-0.68)	0.58(0.48-0.71)	0.45(0.31-0.64)
Low genetic risk			
Unfavorable LF	0.50(0.33-0.77)	0.78(0.62-0.98)	0.62(0.43-0.90)
Intermediate LF	0.39(0.27-0.56)	0.62(0.50-0.75)	0.64(0.46-0.89)
Favorable LF	0.33(0.20-0.53)	0.47(0.35-0.61)	0.55(0.33-0.90)
p value for trend	<0.001	<0.001	<0.001

Abbreviation: CRC, colorectal cancer; HR, hazard ratio; CI, confidence interval; LF, lifestyle.

Cox proportional hazards regression adjusted for age, sex, education, Townsend deprivation index and first 10 genetic principal components.

Supplementary Table 9 CRC risk according to genetic and lifestyle risk stratified by education

	College or university degree (n = 129233)		Others (n = 261132)	
Subgroup	HR (95% CI)	*p* value	HR (95% CI)	*p* value
High genetic risk				
Unfavorable LF	1.00 (ref)		1.00 (ref)	
Intermediate LF	0.73(0.56-0.96)	0.026	0.88(0.75-1.04)	0.136
Favorable LF	0.60(0.44-0.84)	0.002	0.80(0.64-1.00)	0.051
Intermediate genetic risk				
Unfavorable LF	0.86(0.67-1.12)	0.260	0.85(0.73-0.99)	0.043
Intermediate LF	0.57(0.45-0.73)	<0.001	0.65(0.57-0.76)	<0.001
Favorable LF	0.52(0.40-0.69)	<0.001	0.54(0.45-0.65)	<0.001
Low genetic risk				
Unfavorable LF	0.86(0.61-1.19)	0.351	0.64(0.52-0.78)	<0.001
Intermediate LF	0.49(0.36-0.65)	<0.001	0.61(0.51-0.73)	<0.001
Favorable LF	0.43(0.30-0.62)	<0.001	0.45(0.34-0.59)	<0.001
p value for trend	<0.001		<0.001	

Abbreviation: CRC, colorectal cancer; HR, hazard ratio; CI, confidence interval; LF, lifestyle.

Cox proportional hazards regression adjusted for age, sex, education, Townsend deprivation index and first 10 genetic principal components.

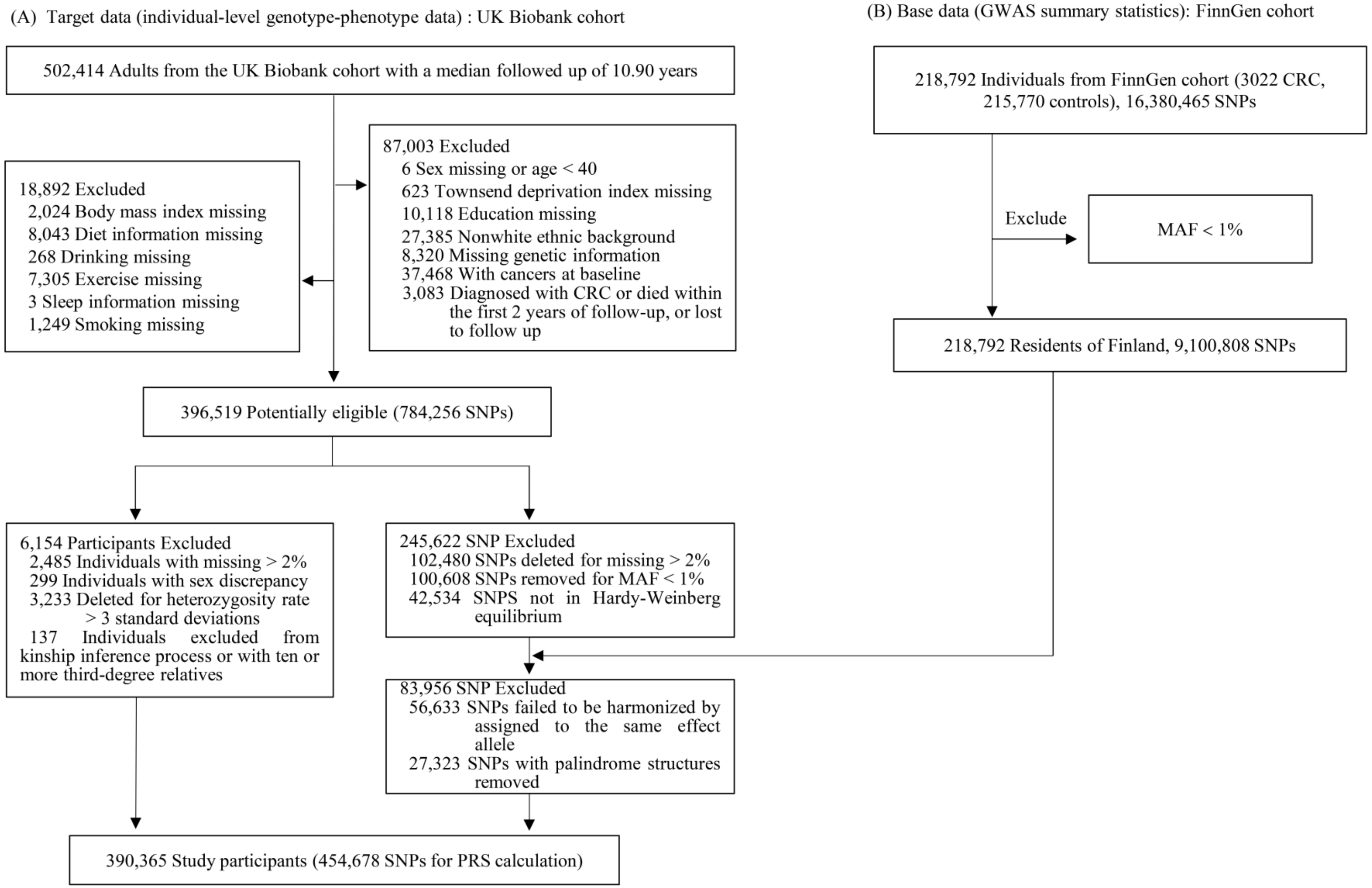

Supplementary Figure 1 Cohort exclusions of the study participants

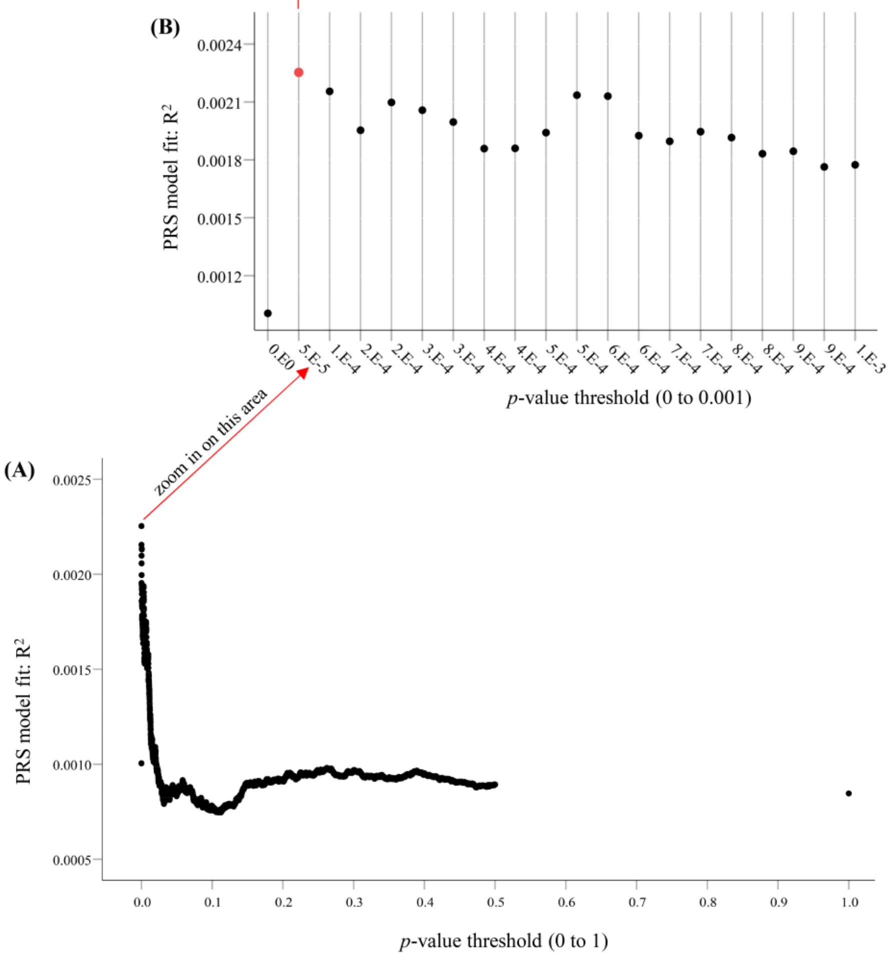

(C)

SNP	CHR	ref	alt	*p*-value	beta	OR
rs4939827	18	T	C	4.48E-08	-0.1461	0.86
rs4464148	18	T	C	2.82E-07	0.1559	1.17
rs7103178	11	C	T	3.92E-07	-0.1589	0.85
rs2001754	11	G	A	3.98E-07	-0.1628	0.85
rs3802842	11	C	A	4.87E-07	-0.1573	0.85
rs2736100	5	C	A	9.10E-07	0.1307	1.14
rs60558873	10	A	G	9.61E-07	0.2706	1.31
rs2965299	19	G	A	1.28E-06	0.1653	1.18
rs961253	20	C	A	2.44E-06	0.1349	1.14
rs7242007	18	G	A	2.56E-06	0.1348	1.14
rs17743102	10	T	G	2.99E-06	0.2618	1.30
rs432266	20	T	C	3.16E-06	-0.1353	0.87
rs12817211	12	C	T	3.97E-06	0.1574	1.17
rs75518588	18	G	A	4.72E-06	0.1508	1.16
rs2193352	10	A	G	5.94E-06	0.1636	1.18
rs115749364	2	G	A	6.64E-06	-0.4155	0.66
rs78362716	12	A	G	8.44E-06	0.1546	1.17
rs7554426	1	G	T	9.96E-06	-0.1253	0.88
rs72831930	6	A	G	1.00E-05	-0.1505	0.86
rs2970517	12	T	C	1.18E-05	0.1336	1.14
rs10511825	9	T	C	1.22E-05	0.1384	1.15
rs16921779	10	C	T	1.30E-05	0.2927	1.34
rs1707931	12	T	C	1.79E-05	0.1193	1.13
rs186582401	16	C	T	2.08E-05	0.2711	1.31
rs10090398	8	G	A	2.08E-05	0.1436	1.15
rs61327167	8	T	C	2.09E-05	0.143	1.15
rs75865824	12	C	T	2.11E-05	-0.2761	0.76
rs35031884	12	C	A	2.15E-05	0.1582	1.17
rs6983267	8	G	T	2.19E-05	-0.1132	0.89
rs12575708	11	C	T	2.29E-05	0.1734	1.19
rs35352860	18	C	T	2.48E-05	-0.1195	0.89
rs6580742	12	C	T	2.50E-05	0.1569	1.17
rs11668951	19	C	T	2.54E-05	0.2856	1.33
rs78720743	6	G	A	2.56E-05	0.2496	1.28
rs118077853	9	C	A	2.61E-05	-0.4019	0.67
rs7508374	19	A	G	2.68E-05	0.1246	1.13
rs4507531	5	G	A	2.68E-05	0.1935	1.21
rs57627107	20	A	G	2.72E-05	-0.1572	0.85
rs28399071	15	G	A	2.99E-05	0.1711	1.19
rs80328255	1	C	T	3.21E-05	0.361	1.43
rs12486257	3	A	C	3.25E-05	0.2198	1.25
rs10505477	8	A	G	3.28E-05	-0.1107	0.90
rs7014346	8	A	G	3.40E-05	-0.1158	0.89
rs73083764	2	A	C	3.43E-05	0.227	1.25
rs7111910	11	A	C	3.43E-05	0.1233	1.13
rs74846031	4	A	G	3.71E-05	0.2992	1.35
rs7310279	12	A	G	3.78E-05	0.151	1.16
rs61922777	12	C	T	3.89E-05	-0.2305	0.79
rs16892766	8	A	C	4.10E-05	0.1664	1.18
rs141738059	9	T	C	4.19E-05	0.4204	1.52
rs6921746	6	G	A	4.22E-05	0.2548	1.29
rs73027931	19	A	G	4.24E-05	0.2752	1.32
rs80255063	16	T	C	4.34E-05	0.2484	1.28
rs6937605	6	C	T	4.35E-05	0.1454	1.16
rs5763178	22	A	G	4.35E-05	-0.1118	0.89
rs12474979	2	G	A	4.67E-05	0.1766	1.19
rs12446169	16	A	C	4.88E-05	0.3846	1.47
rs12269541	10	A	C	4.97E-05	0.1338	1.14
rs9490938	6	C	A	4.98E-05	0.1444	1.16

Supplementary Figure 2 PRSice-2 showing results at broad *p*-value thresholds for PRS predicting CRC (A) Scatter plot at a *p*-value thresholds from 0 to 1; (B) Scatter plot at a p-value thresholds from 0 to 0.001, the red bar indicates the range of *p*-values for which the best-fit PRS was obtained; (C) The specific information of the 59 SNPs contained in the best-fit PRS, when *p*-value < 5×10^{-5}.

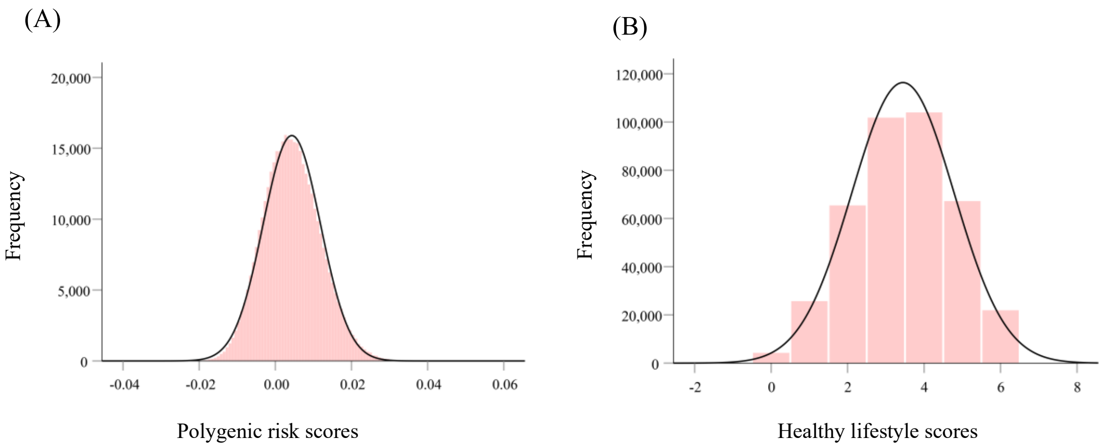

Supplementary Figure 3 Distribution of the polygenic risk scores and healthy lifestyle scores (A) Polygenic risk score for CRC; (B) Healthy lifestyle scores.

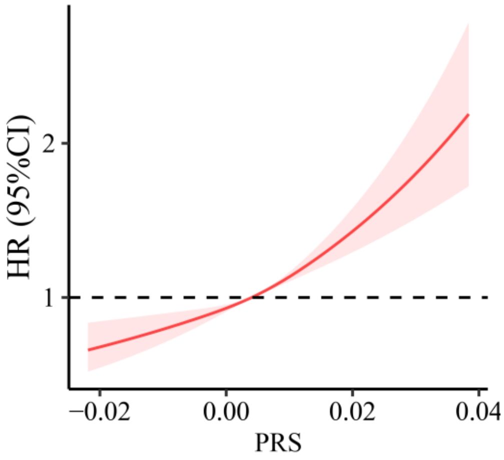

Supplementary Figure 4 Multivariable-adjusted dose-response associations between PRS and CRC risk

The dose-response associations were examined in Cox proportional hazard regression models adjusted for age (years), sex (male, female), education (higher, middle, lower, vocational, other), Townsend deprivation index, and first 10 GPCs at recruitment, based on restricted cubic splines with 3 knots, and the shaded area represents the 95% CI for the dose-response curve. *p* for nonlinearity > 0.05.

Abbreviations: CRC, Colorectal cancer; PRS, Polygenic Risk Scores; HR, hazard ratio; CI, Confidence interval; GPC, genetic principal component.

Weighted lifestyle category	CRC/Controls	HR (95%CI)	HR (95%CI)	p
Unfavorable (quintile 1)	1040/82008	1.00(ref)		
Intermediate (quintiles 2-4)	2359/229818	0.80(0.74-0.86)		<0.001
Favorable (quintiles 5)	691/74449	0.73(0.66-0.80)		<0.001

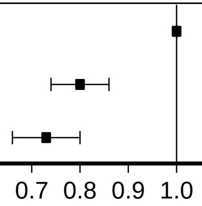

Supplementary Figure 5 Risk of CRC according to weighted lifestyle category

Weighted healthy lifestyle scores were created based on six factors (smoking, drinking, body mass index, diet, exercise, and sleep) according to the formula:

weighted healthy lifestyle scores = $(\beta_{-1} \times \text{factor}_{-1} + \ldots + \beta_{-6} \times \text{factor}_{-6}) \div (\beta_{-1} + \ldots + \beta_{-6}) \times 6$ (1).

The β coefficients of each factor was shown in **Supplementary Table 2**. Each factor was coded as 0,1, and the healthy behavior was defined as 1.

Cox proportional hazards regression adjusted for age, sex, education, Townsend deprivation index and first 10 genetic principal components.

The weighted healthy lifestyle scores were subsequently categorised as: unfavourable (lowest quintile), intermediate (quintiles 2–4), and favourable (highest quintile).

1. Lourida I, Hannon E, Littlejohns TJ, Langa KM, Hyppönen E, Kuzma E, Llewellyn DJ. Association of Lifestyle and Genetic Risk With Incidence of Dementia. JAMA. 2019; 322(5):430-437. doi: 10.1001/jama.2019.9879.

Subgroup		CRC/Controls	HR (95% CI)	Hazard ratio	*p*
High genetic risk					
	Unfavorable LF	232/16467	1.00(ref)		
	Intermediate LF	615/45692	0.96(0.83-1.12)		0.617
	Favorable LF	170/14897	0.81(0.66-0.98)		0.033
Intermediate genetic risk					
	Unfavorable LF	643/49222	0.95(0.81-1.10)		0.468
	Intermediate LF	1342/137992	0.69(0.60-0.80)		<0.001
	Favorable LF	401/44623	0.64(0.55-0.76)		<0.001
Low genetic risk					
	Unfavorable LF	165/16319	0.73(0.60-0.89)		0.002
	Intermediate LF	402/46134	0.61(0.52-0.72)		<0.001
	Favorable LF	120/14929	0.58(0.46-0.72)		<0.001

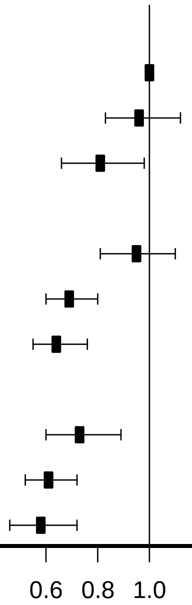

Supplementary Figure 6 Risk of CRC according to genetic and weighted lifestyle categories

Abbreviation: LF, lifestyle; HR, hazard ratio; CI, confidence interval.

Cox proportional hazards regression adjusted for age, sex, education, Townsend deprivation index and first 10 principal components of Ancestry.
